# Obesity pharmacotherapy: current status

**DOI:** 10.17179/excli2014-732

**Published:** 2015-02-20

**Authors:** Parveen Kumar, Uma Bhandari

**Affiliations:** 1 Department of Pharmacology, Faculty of Pharmacy, Jamia Hamdard (Hamdard University)

## ⁯⁯

Dear Editor,

Obesity is a medical condition in which excess body fat has accumulated to such an extent that it may have an adverse effect on health, leading to reduced life expectancy (Haslam and James, 2005[12]). The lifestyle treatments directed at improving diet and physical activity are considered as first line treatment for obesity; however, if these fail, antiobesity medication is recommended. In the past years, numerous drugs have been approved for the treatment of obesity; however, currently, orlistat is the only food and drug administration (FDA) approved drug for long term management of obesity (Table 1(Tab. 1)). (References of Table 1: Elangbam, 2009[9]; Ioannides-Demos et al., 2011[13]; Kang and Park, 2012[14]).

Lipolytic rate in white adipose tissue (WAT) has been positively and negatively correlated with indexes of insulin resistance and WAT *de novo* lipogenesis gene expression. It is a reasonable hypothesis that the difficulty in mobilizing lipids in adipocytes could contribute to increased adiposity and obesity, and thereby reducing the insulin sensitivity (Caminhotto et al., 2014[3]). On the contrary, Girousse et al. (2013[11]) has reported a new mechanism for the regulation of insulin sensitivity, who demonstrated that partial inhibition of lipolysis via reduced action of hormone sensitive lipase (HSL), either by genetic modification or by pharmacological inhibition, reshapes the fatty acid fluxes without increase of fat mass; improving glucose metabolism through cell-autonomous induction of fat cell *de novo* lipogenesis and leading to improved insulin sensitivity in mice. Orlistat is a well-known inhibitor of pancreatic lipase (PL) that is also reported to inhibit HSL; thus inhibiting the stimulated lipolysis (Clifford et al., 2000[5]; Bustanji et al., 2010[2]). It is reported that inhibition of HSL improves lipid profile while reduces plasma glucose (Claus et al., 2005[4]). In this context, inhibition of lipolysis with orlistat provides a mechanism for the decrease plasma free fatty acids (FFA) and improvement in insulin sensitivity. Hence, the control of metabolic activity in WAT stands out as an important therapeutic intervention in the treatment of metabolic diseases (Caminhotto et al., 2014[3]).

Enç et al. (2009[10]) reported that orlistat accelerates gastric emptying and attenuates gastric inhibitory peptide (GIP) release in healthy subjects; that play an important role in the modulation of lipid metabolism, obesity and insulin resistance. GIP, an insulinotropic hormone, is secreted from enteroendocrine upper gut K-cells postprandially. GIP similar to the incretin effect of glucagon-like peptide-1 (GLP-1), stimulates glucose-dependent insulin secretion. By acting on GIP receptors on adipocytes, GIP exhibits insulin mimetic properties such as elevation in glucose uptake, fatty acid synthesis, lipoprotein lipase synthesis, and reduction in glucagon-induced lipolysis; resulting in fat accumulation in adipocytes, obesity and insulin resistance. 

Apart from decreasing insulin resistance, orlistat is reported to increase postprandial GLP-1 levels; thereby enhancing the insulin sensitivity and blunting the postprandial rise in blood glucose in type 2 diabetic patients. Hence, increased GLP-1 levels, which lead to decreased food intake, may also contribute to the weight loss that is associated with the use of orlistat (Damci et al., 2004[6]). Elevated anorectic gut hormones, such as GLP-1 and peptide YY (PYY), play a crucial role in the reduction in food intake. The long-term inhibition of intestinal lipase by orlistat increases the pre-prandial levels of GLP-1 and PYY, independent of body mass changes. Therefore, long-term treatment with orlistat may exert hunger suppressing and insulin sensitizing incretin effect beyond the weight reduction (Olszanecka-Glinianowicz et al., 2013[16]).

Blood glucose control becomes increasingly challenging in the obese patient with type 2 diabetes after the failure of metformin monotherapy (Scheen, 2003[18]; Niswender, 2010[15]). Moderate weight loss is recommended in these patients, with the conjunctive use of weight loss medications. Orlistat is reported to be as effective as metformin in reducing body weight and insulin resistance in obese patients (Sari et al., 2004[17]). As per earlier reports, a moderate weight loss with orlistat treatment resulted in greater improvement in FFA levels and insulin sensitivity in type 2 diabetics (Derosa et al., 2012[7]; Olszanecka-Glinianowicz et al., 2013[16]) as well as obese subjects (Tiikkainen et al., 2004[19]; Derosa et al., 2010[8]). 

With respect to the future prospects point of view, obesity is set to be the world's major cause of morbidity and mortality in the 21st Century. The year 2013 was a landmark for the field of obesity, as in June 2013, the American Medical Association recognizes obesity as a disease (AMA, 2013[1]). The worldwide obesity prevalence continues to increase with a growing demand for effective and safe antiobesity drugs. In the last few years, despite promising results on reduction of body weight, many antiobesity drugs were withdrawn from the market due to serious adverse effects (Kang and Park, 2012[14]). Currently, orlistat is the only FDA approved drug for long term management of obesity. Therefore, there is need for development of more effective and safe antiobesity drugs. Additionally, a lifestyle modification such as diet and exercise need to be focused as a positive approach for treatments for obesity as it remains the cornerstone in the management of obesity. 

## Figures and Tables

**Table 1:**
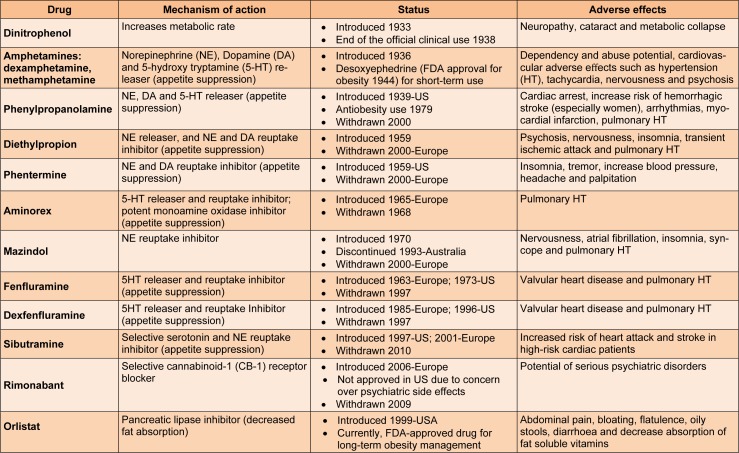
Antiobesity drugs and their current status (Elangbam, 2009; Ioannides-Demos et al., 2011; Kang and Park, 2012)

## References

[1] AMA (American Medical Association) (2013). http://www.amaassn.org/resources/doc/csaph/a13csaph3.pdf.

[2] Bustanji Y, Issa A, Mohammad M, Hudaib M, Tawah K, Alkhatib H (2010). Inhibition of hormone sensitive lipase and pancreatic lipase by Rosmarinus officinalis extract and selected phenolic constituents. J Med Plants Res.

[3] Caminhotto Rde O, Campaña AB, Lima FB (2014). Lipolysis inhibition as therapeutic target in the metabolic syndrome. Arq Bras Endocrinol Metabol.

[4] Claus TH, Lowe DB, Liang Y, Salhanick AI, Lubeski CK, Yang L (2005). Specific inhibition of hormone-sensitive lipase improves lipid profile while reducing plasma glucose. J Pharmacol Exp Ther.

[5] Clifford GM, Londos C, Kraemer FB, Vernon RG, Yeaman SJ (2000). Translocation of hormone-sensitive lipase and perilipin upon lipolytic stimulation of rat adipocytes. J Biol Chem.

[6] Damci T, Yalin S, Balci H, Osar Z, Korugan U, Ozyazar M (2004). Orlistat augments postprandial increases in glucagon-like peptide 1 in obese type 2 diabetic patients. Diabetes Care.

[7] Derosa G, Cicero AF, D'Angelo A, Fogari E, Maffioli P (2012). Effects of 1-year orlistat treatment compared to placebo on insulin resistance parameters in patients with type 2 diabetes. J Clin Pharmol Ther.

[8] Derosa G, Maffioli P, Ferrari I, D'Angelo A, Fogari E, Palumbo I (2010). Orlistat and L-carnitine compared to orlistat alone on insulin resistance in obese diabetic patients. Endocr J.

[9] Elangbam CS (2009). Current strategies in the development of anti-obesity drugs and their safety concerns. Vet Pathol.

[10] Enç FY, Ones T, Akin HL, Dede F, Turoğlu HT, Ulfer G (2009). Orlistat accelerates gastric emptying and attenuates GIP release in healthy subjects. Am J Physiol Gastrointest Liver Physiol.

[11] Girousse A, Tavernier G, Valle C, Moro C, Mejhert N, Dinel AL (2013). Partial inhibition of adipose tissue lipolysis improves glucose metabolism and insulin sensitivity without alteration of fat mass. PLoS Biol.

[12] Haslam DW, James WP (2005). Obesity. Lancet.

[13] Ioannides-Demos LL, Piccenna L, McNeil JJ (2011). Pharmacotherapies for obesity: past, current, and future therapies. J Obes.

[14] Kang JG, Park CY (2012). Anti-obesity drugs: A review about their effects and safety. Diabetes Metab J.

[15] Niswender K (2010). Diabetes and obesity: therapeutic targeting and risk reduction-a complex interplay. Diabetes Obes Metab.

[16] Olszanecka-Glinianowicz M, Dąbrowski P, Kocełak P, Janowska J, Smertka M, Jonderko K (2013). Long-term inhibition of intestinal lipase by orlistat improves release of gut hormones increasing satiety in obese women. Pharmacol Rep.

[17] Sari R, Balci MK, Coban E, Yazicioglu G (2004). Comparison of the effect of orlistat vs orlistat plus metformin on weight loss and insulin resistance in obese women. Int J Obes Relat Metab Disord.

[18] Scheen AJ (2003). Current management strategies for coexisting diabetes mellitus and obesity. Drugs.

[19] Tiikkainen M, Bergholm R, Rissanen A, Aro A, Salminen I, Tamminen M (2004). Effects of equal weight loss with orlistat and placebo on body fat and serum fatty acid composition and insulin resistance in obese women. Am J Clin Nutr.

